# Characterization and Improvement of Heat Resistance of a Polymer-Ceramic Pressure-Sensitive Paint at High Temperatures

**DOI:** 10.3390/s21248177

**Published:** 2021-12-07

**Authors:** Takenori Furuya, Takumi Nakai, Masato Imai, Masaharu Kameda

**Affiliations:** Department of Mechanical Systems Engineering, Tokyo University of Agriculture and Technology, Koganei, Tokyo 184-8588, Japan; t-furuya@st.go.tuat.ac.jp (T.F.); t-nakai@st.go.tuat.ac.jp (T.N.); m-imai@st.go.tuat.ac.jp (M.I.)

**Keywords:** pressure-sensitive paint, flow measurement, unsteady flow, porphyrin, photodegradation, high-temperature environment

## Abstract

Degradation of fast response pressure-sensitive paints (PSP) above room temperature is a serious problem for PSP measurements in high-temperature environments. A standard polymer-ceramic PSP (PC-PSP) composed of platinum(II)-5,10,15,20-tetrakis-(2,3,4,5,6-pentafluorphenyl)-porphyrin (PtTFPP), titania particles and poly(isobutyl methacrylate) (polyIBM) was characterized to elucidate the degradation mechanism. Applying a two-gate lifetime-based method, the PC-PSP has sufficient pressure and temperature sensitivities even at 100 °C, while the luminescence intensity significantly decreases during the test. Subsequent measurements on thermal and photostability as well as luminescence spectra reveal that the main cause of the degradation is the photodegradation of PtTFPP due to direct exposure of the dye molecules to the atmosphere. In order to suppress such degradation, a small amount of urethane resin is added to the dye solution as a simple additional step in the preparation of PC-PSP. The addition of the urethane resin significantly reduces the degradation of the PSP, although its time response is slightly slower than that of the standard PC-PSP.

## 1. Introduction

Pressure-sensitive paint (PSP) is a molecular sensor that uses luminescent dyes emitting fluorescence or phosphorescence whose properties depend on pressure and temperature [1,2]. PSP can be applied to the surface of an object where it is difficult to install sensors or pressure taps, because luminescence is measured non-intrusively with a camera, etc. The development of PSPs with fast response times for unsteady flow field measurement is a major challenge in PSP technology [3,4,5]. The time response of PSP is mostly dominated by gas diffusion in the paint, which can be improved by using porous materials in the paint. The common fast-response PSPs are adsorptive coatings on anodic porous alumina (AA-PSP) [6,7,8] and polymer-ceramic PSPs (PC-PSP) with ceramic fine particles such as silica and titania powders [9,10,11,12,13]. 

Rotor blades are a suitable object to which to apply fast response PSPs. In this paper, we focus on the application of fast-response PSP to compressors [14,15,16,17]. From the PSP measurement point of view, a remarkable characteristic of compressors is that the temperature of the measurement environment exceeds 100 °C. Previous studies have demonstrated that measurements can be made, but it is not clear whether the accuracy is sufficient to evaluate the performance of the compressor. 

The degradation of PSP at temperatures above room temperature is one of the factors that have reduced the measurement accuracy of PSP in previous studies [17,18,19]. Kitamura et al. [17] evaluated AA-PSP using a ruthenium complex (Ru(dpp)) as PSP dye, while Li et al. [18] and Peng et al. [19] evaluated PC-PSP using a porphyrin (PtTFPP). All of them show that a decrease in luminescence intensity occurs, which is not seen at room temperature. Peng et al. [19] speculated that the degradation was caused by glass transition of the polymer whose temperature was below that of the measurement environment. However, similar degradation also occurs in the AA-PSP without the polymer. The luminescence properties of PtTFPP did not change after it was dissolved in a solvent and exposed to high temperature (230 °C) [20]. It is necessary to clarify the degradation mechanism exactly. 

Peng et al. [19] developed a PC-PSP using PtTFPP, mesoporous silica and polystyrene with a high glass transition temperature. They reported that this PSP could be used in a temperature environment of 140 °C. A luminescent lanthanide coordination polymer is also being considered as a new thermo-stable pressure sensor whose decomposition temperature is 400 °C [21]. However, if it is possible to improve their heat resistance, it is more practical to apply additional treatment to common PSPs instead of completely renewing the material. 

**Figure 1:**
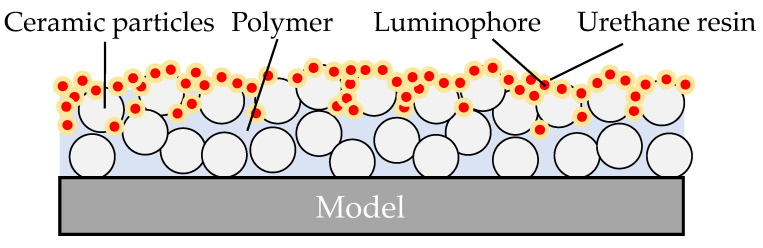
PC-PSP with added Urethane resin.

In this study, we derive a reliable explanation for the degradation of standard PC-PSPs at high temperatures. For this purpose, we investigate in detail not only the luminescence intensity, pressure and temperature sensitivities, and glass transition temperature, but also the luminescence spectra. We then test a thin layer of urethane resin with dissolved dye on the top of the PC-PSP to improve its heat resistance as shown in Figure 1. We show that the heat resistance of PC-PSPs can be improved by the addition of urethane resin, which inhibits the dye molecules from being exposed to ambient gases directly. We evaluate the changes in pressure and temperature sensitivities, thermal and photodegradation of luminescence, and temporal response with the amount of urethane resin added. 

## 2. Materials and Methods

### 2.1. Materials and Preparation

We prepared the polymer-ceramic pressure-sensitive paint (PC-PSP) according to the recipe by Sugioka et al. [12] as a standard sample of fast-response PSP. The PC-PSP is composed of luminescent dye, polymer and ceramic particles. We used poly(isobutyl methacrylate) (polyIBM, Sigma-Aldrich, St. Louis, MO, USA) as the polymer, titanium-oxide (titania) particles (typical diameter 15 nm, MT-150EX, Tayca, Osaka, Japan) as the ceramic particles, and toluene (Fujifilm Wako Pure Chemical, Osaka, Japan) as the solvent for the polymer. The total mass of the polymer and titania particles was 7.14 g in 30 mL of solvent, and the mass fraction of titania particles was 90 wt% of the total solid mass. For the dye solution, we used 30 mg of platinum(II)-5,10,15,20-tetrakis-(2,3,4,5,6-pentafluorphenyl)-porphyrin (PtTFPP, Frontier Scientific, Logan, UT, USA) as the luminescent dye, and 30 mL of toluene as the solvent. In addition, 1.8 g of dichloromethane (Fujifilm Wako Pure Chemicals, Osaka, Japan) was added to enhance the dissolution of the dye. 

We employed the following procedure to apply the PC-PSP to the surface of the object. First, the polymer solution containing ceramic particles and the dye solution were prepared separately. After stirring for 12 h, the polymer solution was painted onto an aluminum plate (20 mm × 20 mm) using a spray gun. The plate was dried in a vacuum drying oven at room temperature for at least 12 h. Then, the dye solution, which was stirred for more than 1 h, was applied directly on top of the binder using the spray gun. 

Next, we tested whether the heat resistance of PC-PSP would be improved by adding a small amount of urethane resin to the dye solution. We used a two-component polyurethane paint (Urethane clear coat, Cromax HC-7776S and activator, Cromax 7775S. Axalta Coating Systems, Philadelphia, PA, USA) as the material, which was formulated at a volume ratio of 4 clear coat to 1 activator. The mass of urethane resin to be added to 30 mL of dye solution was 0.5 g or 1.0 g. After stirring the materials other than the activator for at least one hour, the activator was added and stirred for another five minutes, after which the dye solution with urethane resin was applied. 

In addition, we prepared a polymer-based PSP to compare the differences in various properties depending on the binder material. A mixture of 30 mg of PtTFPP, 20 mL of toluene, 1.8 g of dichloromethane, and 20 mL and 5 mL of polyurethan clear coat and activator, respectively, was used as the paint. This mixture was applied with a spray gun to an aluminum plate that had been previously coated with a white paint (Kampe Hapio, Kansai Paint, Osaka, Japan) and had been dried in the vacuum drying oven at room temperature for at least 12 h. 

### 2.2. Performance Test

#### 2.2.1. Pressure and Temperature Sensitivity

**Figure 2:**
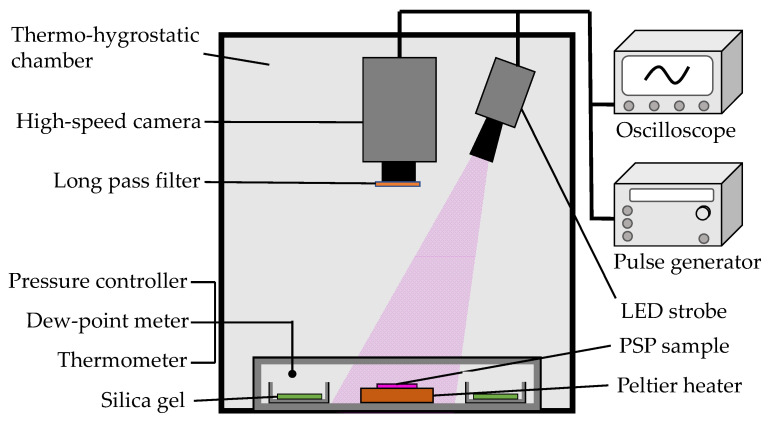
Schematic diagram of the variable pressure chamber.

The pressure and temperature sensitivity of PSP was evaluated using a variable pressure chamber shown in Figure 2. The pressure chamber was placed in a thermo-hygrostatic chamber (PR-3J, Espec, Osaka, Japan) where temperature and humidity could be controlled separately. The PSP sample was placed on a thermoelectric (Peltier) plate heater (KLT-2R, Yamaki, Machida, Japan) to control the temperature of the sample. An optical glass window was located at the top of the chamber to illuminate and observe the sample. A precision pressure controller (Druck PACE5000, Baker Hughes, Houston, TA, USA) was used to set the pressure in the chamber, and dry air was used as the gas in the chamber. In order to reduce the effect of humidity on the properties of porous PSPs [22], A-type silica gel (Fujifilm Wako Pure Chemical, Osaka, Japan) was placed inside the chamber to maintain low humidity. 

The optical setup for the PSP measurement is also displayed in Figure 2. A violet LED illuminator (IL106, Hardsoft Microprocessor Systems, Krakow, Poland) was used as a light source for PSP dye excitation, and a high-speed video camera (AX-200, Photron, Tokyo, Japan) was used as a detector of PSP signals. The emission wavelength of the violet LED (CBT-120-UV-X31-L400-22, Luminus Devices, Sunnyvale, CA, USA) was 400–410 nm. The driving current for LED was set to 32 A. The camera was equipped with a lens (35 mm f/1.4, Nikon, Tokyo, Japan) and a long-pass filter (O58, Hoya, Tokyo, Japan) to eliminate light below 580 nm, including excitation light. A pulse generator (9614, Quantum Composers, Bozeman, MT, USA) was used to control the light source and camera.

We employed a lifetime-based method to evaluate the pressure and temperature sensitivity of the PSP. The time-resolved PSP emission depends on the temperature and the oxygen concentration around the PSP dye [1]. In actual measurements using a camera, the two-gate method is commonly used instead of the time-resolved measurement of luminescence intensity [2,4,23]. In the two-gate method, the intensities are captured at two different times during a single dye emission and quenching process, and the pressure and temperature are calculated from the ratio of the two intensities. The use of the intensity ratio is very advantageous for PSP measurements at high temperatures. This is because, as shown in a previous study [19] and later in this study, the PSP luminescence intensity decreases rapidly when the excitation is continued at high temperature. This degradation is so great that it makes it impossible to employ the standard intensity-based method of using intensities measured at distant times as reference values. 

**Figure 3:**
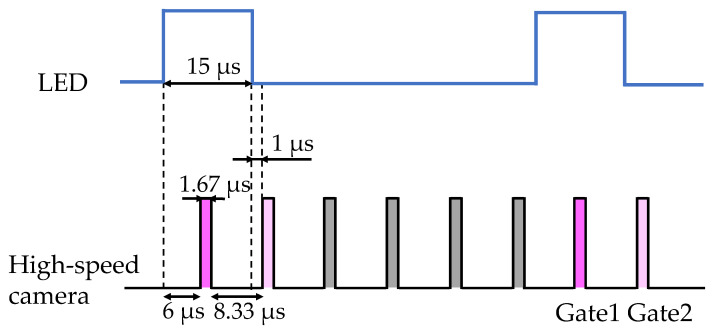
Time sequence of LED and high-speed camera.

We adopted the imaging sequence as shown in Figure 3, which would be directly applied to an actual measurement for turbomachinery. For each pulse of excitation light, a total of six images were taken; the first one (gate 1) during the excitation light illumination, the second one (gate 2) just after the light was turned off, and the third to sixth images when the dye was completely quenched. The duration of the pulsed light was 15 µs. The exposure time of each image was set to 1.67 µs. The first image during the light illumination was taken at 6 µs after the pulsed light was turned on. The second image was taken at 1 µs just after the light was turned off. The third to sixth images were taken at intervals of 8.33 µs. These dark images were used in the subtraction process to reduce the background noise of the high-speed camera. We acquired 1500 images for 250 consecutive pulsed light shots, and the integrated average of the images taken at each timing was used for determination of pressure and temperature sensitivity. 

The pressure and temperature sensitivities were measured in the range of 20 kPa to 180 kPa for pressure and 20 °C to 100 °C for temperature. The pressure and temperature were set every 20 kPa and 20 °C, respectively. Initially, the temperature was kept at 20 °C and the pressure was varied from 20 kPa to 180 kPa. Then, the temperature was increased in increments from 40 °C to 100 °C, and the pressure was varied at each temperature. Finally, the data were measured at 20 °C again, and the pressure and temperature sensitivities were compared before and after heating and excitation light illumination at the same temperature (20 °C). 

#### 2.2.2. Thermal and Photostability

The changes in the luminescence intensity of PC-PSP were obtained using the experimental setup shown in Figure 2, where the temperature of the samples was kept constant for a long period. The temperature of the Peltier heater was set to 150 °C and the PSP sample was opened to the atmosphere. The intensity image started to be captured 20 min after the Peltier heater started its operation, and the images were captured every hour during the 6 h of continuous heating. The intensity data were obtained from the average of 250 consecutive images of gate 1 taken in the same sequence as in Figure 3. 

Photostability was evaluated by measuring the change in luminescence intensity of the PSP samples under continuous excitation using the experimental setup shown in Figure 2. The pressure in the chamber was kept constant at 100 kPa. The temperature of the sample was set to 20°C and 100°C. The distance from the LED to the PSP sample was set to about 30 cm. The output of the LED light was measured simultaneously with a photodiode sensor (C6386-01, Hamamatsu Photonics, Hamamatsu, Japan).

#### 2.2.3. Luminescence Spectra

The luminescence spectra were evaluated using the experimental setup shown in Figure 2. A spectrometer (Flame-T, Ocean Insight, Orlando, FL, USA) equipped with a long-pass filter and optical fiber was used to measure the luminescence spectra. In this experiment, the LED was set to the continuous illumination mode and the output current was set to 3 A, which was weaker than other experiments using the pulse mode. The pressure was fixed at 100 kPa, and the temperature was varied from 20 °C to 100 °C in 20 °C steps to obtain the luminescence spectra. 

#### 2.2.4. Luminescence Intensity

The change in luminescence intensity of PC-PSP due to the addition of urethane resin was evaluated using the experimental setup shown in Figure 2. The intensity was measured at a temperature of 100 °C and a pressure of 100 kPa. To reduce the effect of non-uniformity of excitation light, the positions of PSP samples and LED light sources were fixed precisely. Five samples made with the same recipe were prepared, then the average values of their luminescence intensities were used as the data for comparison. 

#### 2.2.5. Frequency Response

**Figure 4:**
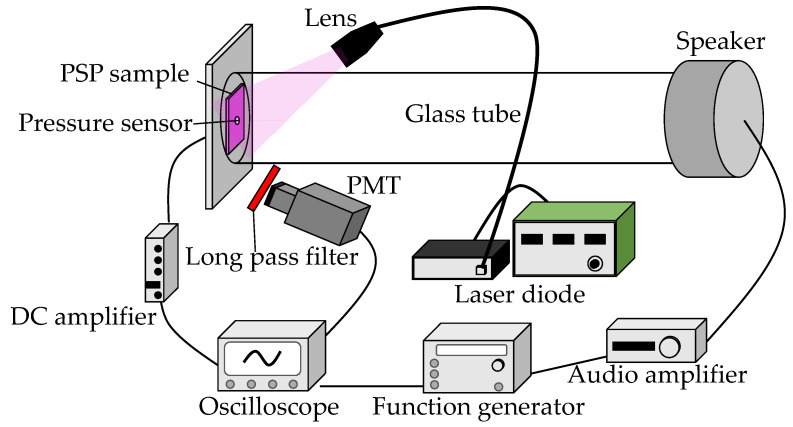
Schematic diagram of acoustic resonance tube.

The frequency response was evaluated using an acoustic resonance tube shown in Figure 4. The resonance tube was a transparent glass tube which had a length of 500 mm and an inner diameter of 25 mm. A loudspeaker (D1405, Fostex, Tokyo, Japan) was placed at one end of the tube, while a PSP sample and a semiconductor pressure transducer (XCQ-093-5D, Kulite Semiconductor Products, Leonia, NJ, USA) were placed at the other end. We drilled a hole in the PSP sample and mounted the transducer in the hole flush with the surface of the sample. The loudspeaker was driven by the signal of a function generator (1930A, NF Corporation, Yokohama, Japan) amplified by an audio amplifier (R-SE7, Kenwood, Yokohama, Japan) to generate simple tones in the range of 0.6 kHz to 3.0 kHz. As it was illuminated with excitation light from a violet laser diode (RV-1000, Ricoh Optical Industries, Hanamaki, Japan), the PSP emission was captured by a photomultiplier tube (PMT, H6780-20, Hamamatsu Photonics, Hamamatsu, Japan) with a long-pass filter (O58, Hoya, Tokyo, Japan) attached to the front. The emission wavelength of the violet laser diode was 400-410 nm. The output signals of the PMT and the pressure sensor amplified by a DC amplifier (AM32, Unipulse, Koshigaya, Japan) were simultaneously recorded by a 16-bit digital oscilloscope (GR-7000, Keyence, Osaka, Japan). 

The two signals were transformed into frequency domain data by fast Fourier transform (FFT) to extract the phase difference between the two signals at the driving frequency of the speaker. The noise of the detector may affect the frequency response [13]. We adopted phase difference for our evaluation because gain plots are particularly subject to noise in our experience. To further reduce the impact of noise on the evaluation, we used a large number of data points (2^16^) to calculate the frequency response. The signal-to-noise ratio (SNR) of the PSP signal detected by the PMT in the frequency domain was about 47 dB, which is large enough to identify the frequency response. 

Next, we determined the characteristic frequency at which the phase delay exhibits –18 deg. This is the frequency at which the inverse of its angular frequency coincides with the time constant of gas diffusion, according to the PSP frequency response theory based on gas diffusion [24]. Five measurements were made on a single sample, and the average of the phase delays obtained from the measurements was used as data for comparison. 

### 2.3. Glass Transition of Polymers

A differential scanning calorimetry (DSC) system (DSC Q200, TA Instruments, New Castle, DE, USA) was used to perform thermal analysis of the polymers. DSC is a technique that measures the level of heat required to raise the temperature of a sample for a certain period of time. The glass transition temperature (*Tg*) was estimated from the shift of the baseline. 

## 3. Results and Discussion

### 3.1. Characterization of Standard PC-PSP

#### 3.1.1. Pressure and Temperature Sensitivity

First, the changes in pressure and temperature sensitivity of PC-PSPs caused by heat treatment without illumination were investigated in detail. In this heat treatment, the PSP samples were placed in a vacuum oven for 12 h at a temperature of 150 °C and an absolute pressure of 30 kPa.

**Figure 5:**
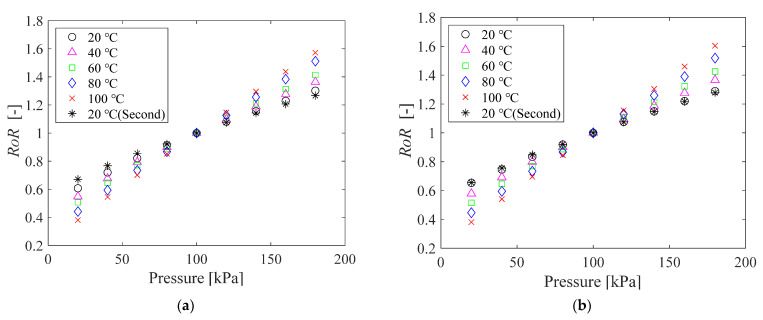
The relationship between the pressure and the *RoR* of PC-PSP (**a**) before and (**b**) after the heat treatment.

The pressure sensitivity of the PC-PSPs before and after the heat treatment was measured according to the procedure described in Section 2.2.1. Figure 5 shows the relationship between the pressure and the ratio-of-ratios (*RoR*) before (Figure 5a) and after (Figure 5b) the heat treatment. The *RoR* is defined as the ratio between the intensities at gate 1 (*S*_1_) and gate 2 (*S*_2_) (see Figure 3) at the reference pressure (*p* = 100 kPa) normalized by the ratio at the run condition, which is

(1) $$RoR=\frac{{\left(S_{2}/S_{1}\right)}_{\mathrm{ref}}}{{\left(S_{2}/S_{1}\right)}_{\mathrm{run}}}.$$

The *RoR* represents the ratio of the luminescence lifetimes in the two states, similar to the intensity ratio (*I*_ref_/*I*_run_) in the intensity-based PSP measurement [4].

**Figure 6:**
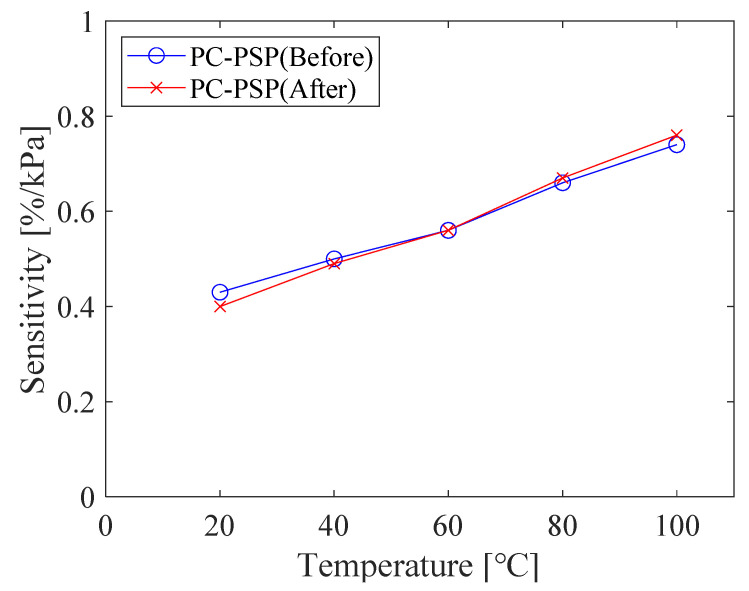
Pressure sensitivity of PC-PSP at different temperatures before and after the heat treatment.

Figure 5 shows that the relationship between *RoR* and pressure at each temperature is almost linear, regardless of whether heating is applied or not. The slope determined by linear approximation increases with increasing temperature, as shown in Figure 6. This tendency is reasonable because the pressure sensitivity of PSP increases with the gas diffusivity in the PSP binder [1], which, in porous material, increases with temperature [8]. Note that the considerable decrease in the pressure sensitivity at high temperature presented by Peng et al. [19] was not observed in this study. 

Focusing on the sensitivity at a temperature of 20 °C, the value slightly decreases during the calibration test; it is 0.43%/kPa for the first time and 0.37%/kPa for the second time before the heat treatment, and 0.40%/kPa for the first time and 0.39%/kPa for the second time after the heat treatment. Nevertheless, the effect of the heat treatment is minor in the lifetime-based PSP measurement, given the fact that the pressure sensitivity at each temperature is almost constant, irrespective of the 12-hour heat treatment.

**Figure 7:**
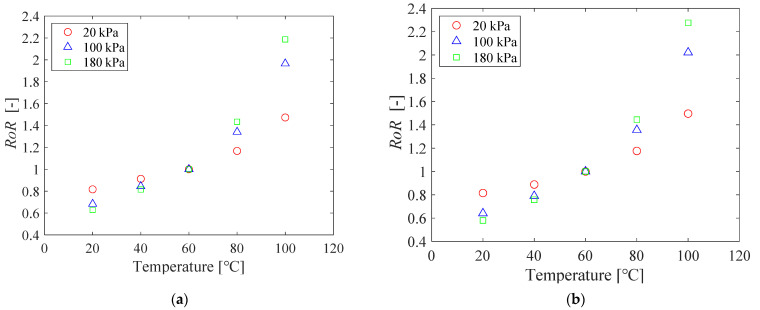
The relationship between the temperature and the *RoR* of PC-PSP (**a**) before and (**b**) after the heat treatment.

The temperature sensitivity of the PC-PSP for the two samples before and after the heat treatment is shown in Figure 7. The two calibration curves are both nonlinear, and the slope increases with increasing pressure. The temperature sensitivity of the heat-treated sample is slightly greater than that of the non-heat-treated sample. The temperature correction is always necessary for PC-PSP measurements based on the lifetime-based method.

#### 3.1.2. Thermal and Photostability

We paid attention to the change in intensity at high temperatures. Table 1 shows the intensity change during the sensitivity test shown in Figure 5. Comparing the luminescence intensity of gate 1 for the first and second measurements at 20 °C and 100 kPa, the intensity decreases to 43.0% before the heat treatment, and 37.6% after the heat treatment, respectively. The PSP was illuminated with excitation light for about 22.5 min during the test, which was from the start of the first measurement at 20 °C to the end of the second measurement at 20 °C. These decreases in the intensity suggests that severe degradation of PC-PSP occurs in high-temperature conditions during the calibration test.

**Figure 8:**
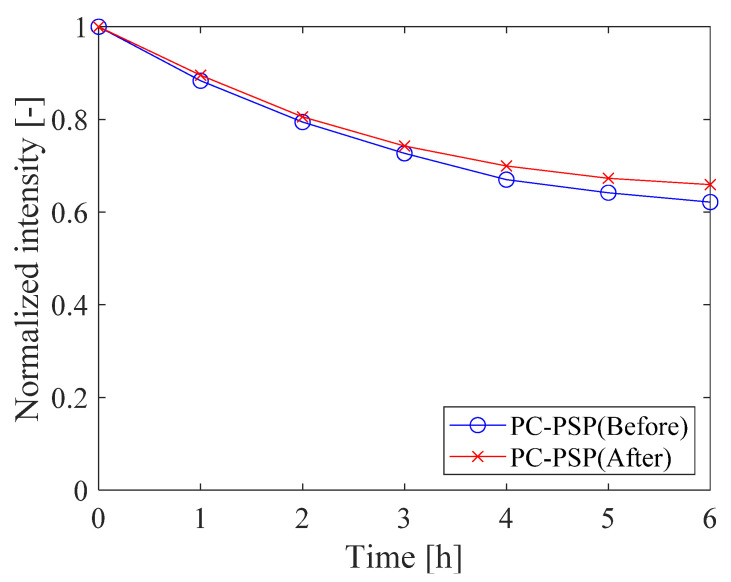
Thermal stability of PC-PSP before and after the heat treatment.

Figure 8 shows the change in intensity of PC-PSP during heating. In this test, the luminescence intensity of the two samples before and after the heat treatment was measured by illuminating the excitation light for 30 s once per hour with the Peltier heater at 150 °C and the pressure open to the atmosphere. After 6 h of heating, the luminescence intensities of the samples before and after the heat treatment decreased to 0.63 and 0.66, respectively. Note that we illuminated the sample for 3.5 min during this test. Therefore, it is not possible to conclude that this decrease in intensity is due to thermal degradation. 

**Figure 9:**
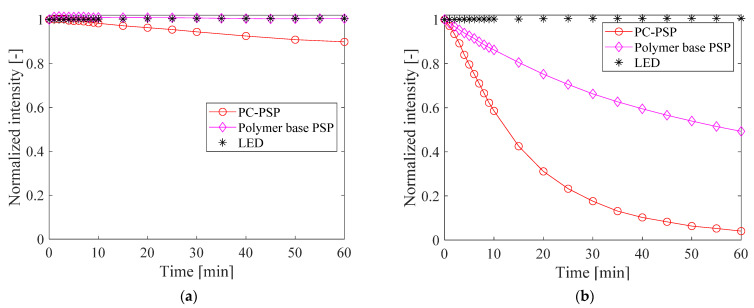
Photostability of PC-PSP after the heat treatment at (**a**) 20 °C and (**b**) 100 °C.

The photostability of the PC-PSP after the heat treatment is shown in Figure 9. The pressure was set to 100 kPa and the temperature was set to 20 °C and 100 °C. The polymer-based PSPs were also evaluated for comparison. The results at 20 °C show that the luminescence intensity of PC-PSP decreased to 0.90 after 1 h of continuous exposure to excitation light. On the other hand, no photodegradation is observed for the polymer-based PSP. At 100 °C, the luminescence intensity of PC-PSP decreases to 0.04 after 1 h of excitation light illumination, while that of the polymer-based PSP is 0.49 after illumination. At 100 °C, the photodegradation of the dye would be accelerated. In particular, the PC-PSP would be more subject to photodegradation than polymer-based PSPs because the dye adsorbed on the binder surface is directly exposed to ambient air. The combination of titania particles and dye is often used for the degradation of organic materials. In general, porphyrins are highly photostable, but at high temperatures, the dye itself is expected to undergo photo-oxidation due to coexistence with titania particles [25]. 

Such a rapid decrease in luminescence intensity at high temperatures makes it difficult to measure the pressure correctly using the intensity-based method. The lifetime-based method should be used if PC-PSP is used at high temperatures.

#### 3.1.3. Luminescence Spectra

**Figure 10:**
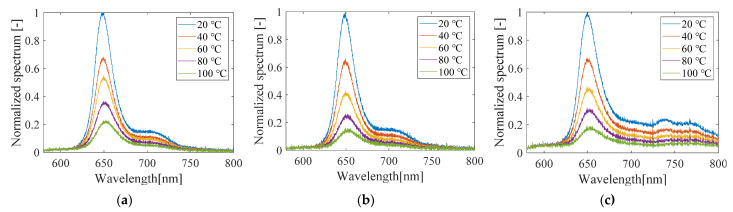
Luminescence spectra of PC-PSP (**a**) before and (**b**) after the heat treatment and (**c**) after photodegradation.

Figure 10 shows the luminescence spectra of the PC-PSP samples with and without the heat treatment, and after light illumination at high temperatures. The light-illuminated sample shown in Figure 9b was illuminated at 100 °C for 1 h. There is almost no change in the luminescence spectra with or without the heat treatment alone. This indicates that the structure of PtTFPP is not changed by heating at 100 °C alone. In fact, no change in the luminescence spectra occurred in an experiment in which the luminescent dye PtTFPP was dissolved in a solvent and heated at 230 °C for 3 h [20]. In contrast, the luminescence spectra of PC-PSP after light illumination differs significantly from the other samples in the wavelength range above 720 nm, although the dominant peak remains at 650 nm. These results indicate that the significant degradation of PC-PSP at high temperature is due to the destruction of part of the dye structure by light [25].

### 3.2. Glass Transition Temperature of Polymers

**Figure 11:**
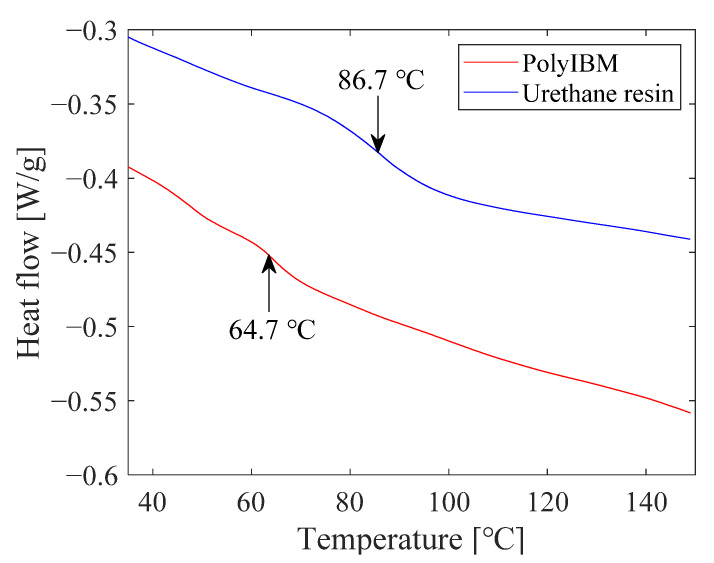
Thermogram of polyIBM and urethane resin.

The thermograms of polyIBM and urethane resin by DSC are shown in Figure 11. The urethane resin was mixed with two components, cured, and then heated at 150 °C in a vacuum oven for 12 h. The shift of the baseline caused by the glass transition occurs at about 64.7 °C for polyIBM and at about 86.7 °C for urethane resin. In addition to these samples, we also performed thermal analysis of the polymer doped with PtTFPP and heat-treated, as well as photodegraded (100 °C, 1 h) samples. The glass transition temperatures of all the samples are summarized in Table 2. The glass transition temperatures of both polymers are increased by the addition of the dye. After photodegradation, the glass transition temperature of polyIBM remains unchanged, while that of urethane resin significantly decreases.

The cause of the significant decrease in *Tg* in urethane resin is unclear. In general, UV-irradiated polyurethane coatings exhibit a decrease in free volume after UV-irradiation by the formation of free radicals due to photochemical processes [26]. However, in the present experiment, the glass transition temperature is significantly lowered. This implies that PtTFPP has an inhibitory effect on photodegradation. In fact, it has been shown that luminescent dyes act as UV absorbers, which reduce the photodegradation of plastics [27]. 

Here we look back at the pressure sensitivity shown in Figure 5. A closer look at the change in the slope of the sample before the heat treatment shows that the slope is larger when the temperature is raised from 60 °C to 80 °C than at a temperature below 60 °C. It is known that the pressure sensitivity of PSP increases with the gas diffusivity [1]. If the temperature exceeds *Tg*, the free volume between polymer chains increases, which increases the gas diffusivity within the polymer [28]. This effect is probably the reason why the increase in pressure sensitivity shown in Figure 3 from 60 °C to 80 °C is slightly larger than that before 60 °C. However, the effects of glass transition on the polymer do not play a major role in the lifetime-based PSP measurement. 

### 3.3. Characterization of PC-PSP with Added Urethane Resin

In this subsection, we show the results of the characteristic test of PC-PSP, in which urethane resin was added. As described in Section 3.1, when PC-PSPs are illuminated with excitation light at high temperatures, a significant decrease in luminescence intensity occurs due to degradation. Even if the lifetime method is used, the significant decrease in luminescence intensity will have a negative impact on the measurements. Therefore, as a means to increase the heat resistance of PC-PSPs, we considered the addition of urethane resin to the dye solution. 

We applied the heat treatment to the PSP samples before the test. For the heat treatment, the samples were placed in a vacuum oven for 12 h at a temperature of 150 °C and an absolute pressure of 30 kPa. 

#### 3.3.1. Pressure and Temperature Sensitivity

**Figure 12:**
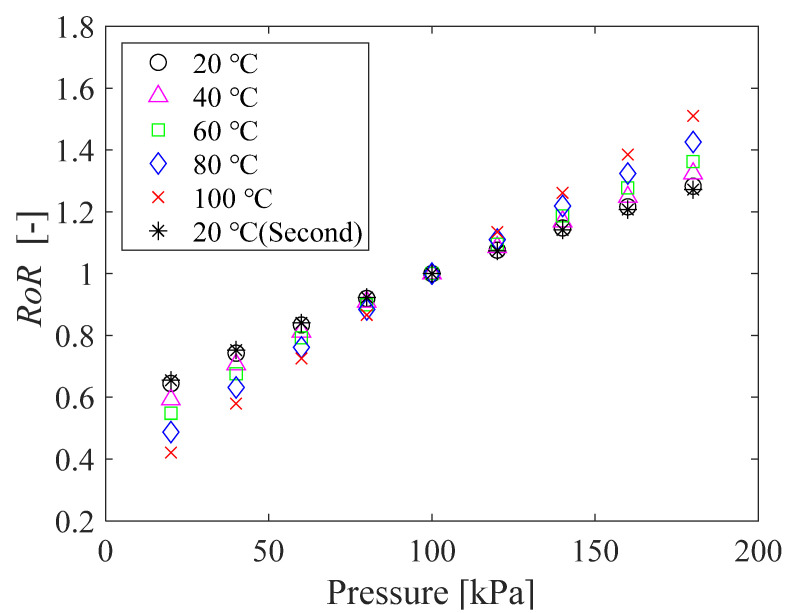
The relationship between the pressure and the *RoR* of PC-PSP with 0.5 g of urethane resin.

**Figure 13:**
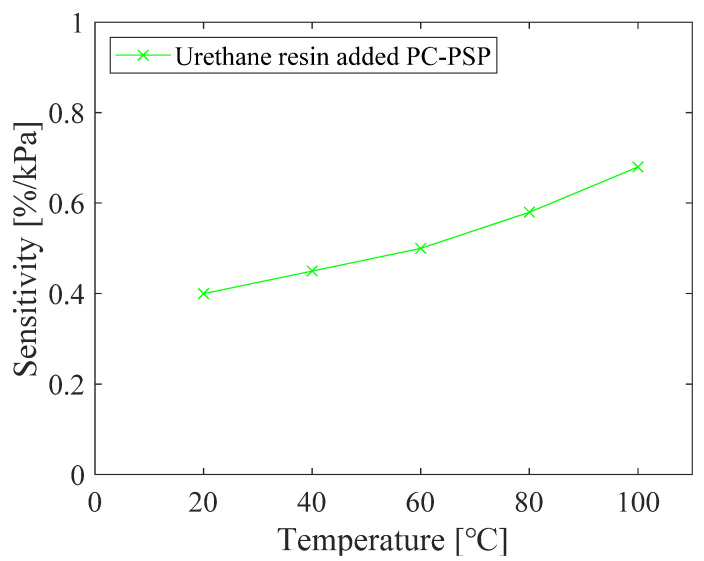
Pressure sensitivity of PC-PSP with 0.5 g of urethane resin at different temperatures.

The relationship between pressure and *RoR* of PC-PSP with 0.5 g of urethane resin is shown in Figure 12. The calibration curve is linear, and the slope of the curve increased with increasing temperature. Figure 13 shows the pressure sensitivity of the PC-PSP obtained by linear approximation of the calibration data at each temperature shown in Figure 12. The pressure sensitivities of the two tests at 20 °C are 0.40%/kPa and 0.38%/kPa, respectively, which are almost the same. Compared to the standard PC-PSP without urethane resin shown in Figure 3, the pressure sensitivity is decreased. Focusing on 100 °C, the pressure sensitivity decreases from 0.76%/kPa to 0.68 %/kPa. This may be due to the decrease in gas permeability around the dye by the addition of urethane resin. On the other hand, the glass transition of the polymer has only a small effect on the pressure sensitivity. From 60 °C to 80 °C, the increase in pressure sensitivity is slightly larger than that below 60 °C. This is probably because the temperature exceeds the glass transition temperature of PolyIBM (*Tg* = 65 °C). The rate of increase of pressure sensitivity with a temperature above 80 °C is larger than that of standard PC-PSP. This is thought to be because the temperature is higher than *Tg* of the urethane resin. 

**Figure 14:**
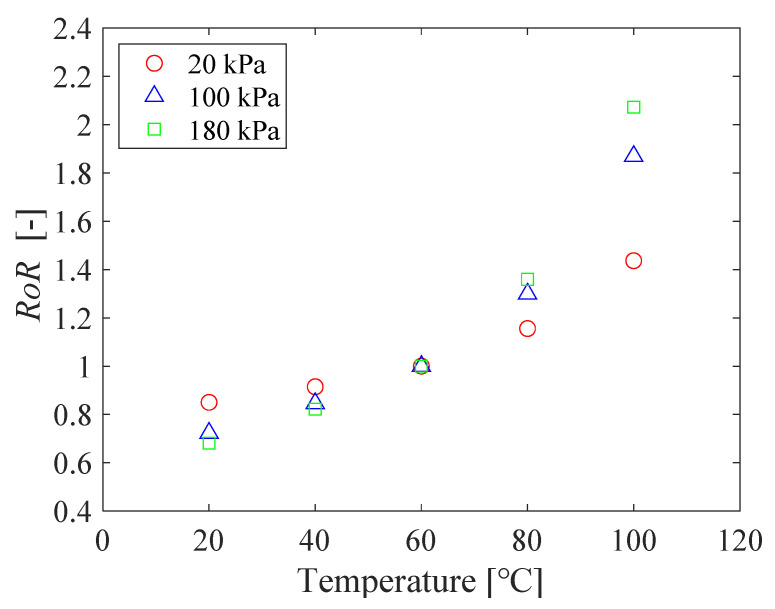
The relationship between the temperature and the *RoR* of PC-PSP with 0.5 g of urethane resin.

The relationship between temperature and *RoR* of PC-PSP with urethane resin is shown in Figure 14. The calibration curves are all nonlinear, and the temperature sensitivity increases with increasing pressure. The temperature sensitivity of the PC-PSP with urethane resin is decreased compared to the standard PC-PSP.

#### 3.3.2. Thermal and Photostability

**Figure 15:**
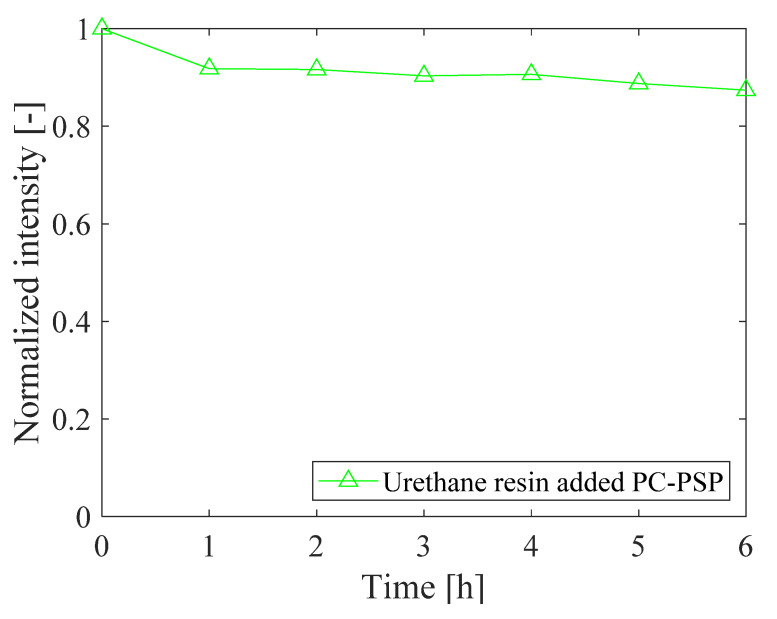
The thermal stability of PC-PSP with 1.0 g of urethane resin.

The thermal stability of PC-PSP with 1.0 g of urethane resin is shown in Figure 15. After 6 h of heating at a temperature of 150 °C and under atmospheric pressure, the luminescence intensity decreases to 0.87 times the initial value. However, this value is much larger than the value shown in Figure 8 for the standard PC-PSP sample (0.66).

**Figure 16:**
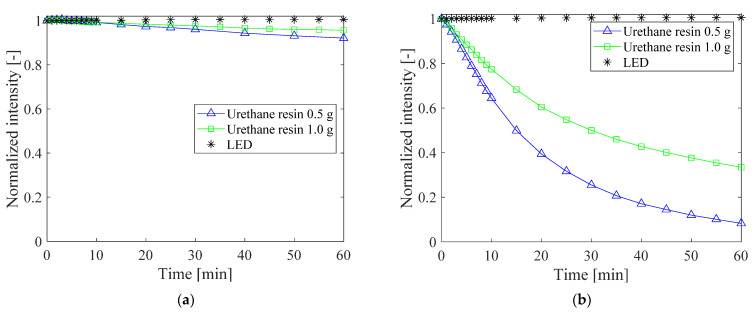
Photostability of PC-PSPs with added urethane resin at (**a**) 20 °C and (**b**) 100 °C.

The photostability of PC-PSP with added urethane resin is shown in Figure 16. The samples were exposed to excitation light for 1 h continuously. The pressure was set to 100 kPa, and the temperature was set to 20 °C or 100 °C. The photodegradation is further suppressed as the amount of urethane resin increases. At 20 °C, the luminescence intensity of PC-PSPs with 0.5 g and 1.0 g of urethane resin decreases to 0.92 and 0.96 times the initial value, respectively, after 1 h of illumination. At 100 °C, the luminescence intensities with 0.5 g and 1.0 g of urethane resin are 0.08 and 0.33 times the initial values, respectively. This suppression of photodegradation is probably due to the interaction between the dye molecules and the surrounding gas, which is reduced by the addition of the urethane resin.

#### 3.3.3. Luminescence Spectra

**Figure 17:**
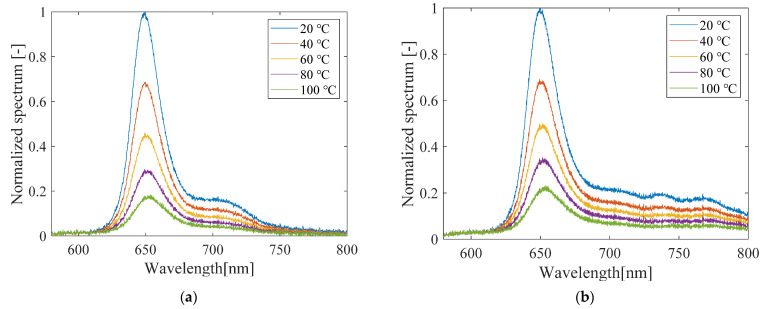
Luminescence spectra of PC-PSP with 1.0 g of urethane resin (**a**) after the heat treatment and (**b**) after photodegradation.

The luminescence spectra of PC-PSP with 1.0 g of urethane resin measured with and without light illumination are shown in Figure 17. The luminescence spectra of the urethane-doped PC-PSP before light illumination are almost the same as those of the PC-PSP without urethane resin shown in Figure 10b. It suggests that the addition of urethane resin does not affect the structure of PtTFPP molecules. For the luminescence spectra of the sample after light illumination, the power in the wavelength longer than 720 nm was smaller than that of the standard PC-PSP shown in Figure 10c. This indicates that the addition of urethane resin suppresses the photodegradation.

#### 3.3.4. Increase in Luminescence Intensity

**Figure 18:**
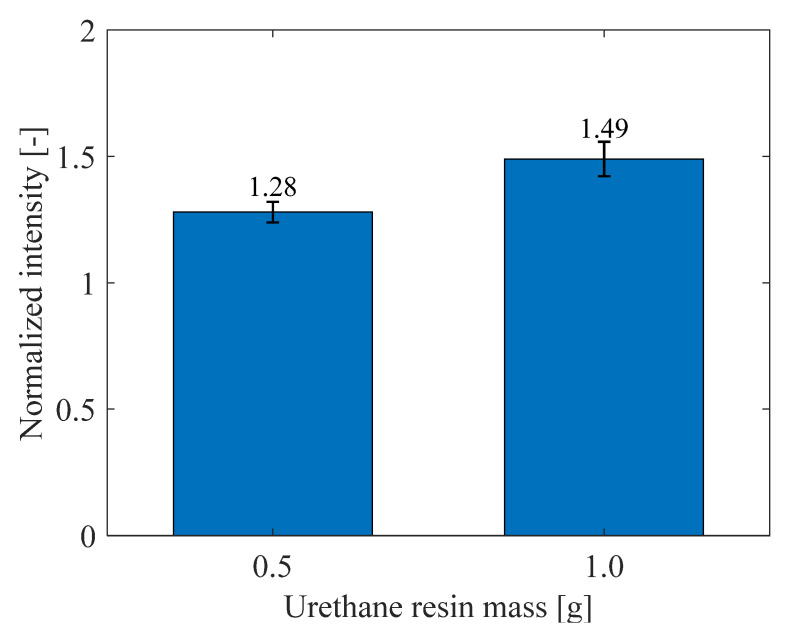
Normalized luminescence intensities of PC-PSPs with different amounts of urethane resin.

The luminescence intensities of PC-PSPs with different amounts of urethane resin are shown in Figure 18 where the intensities of PC-PSPs doped with urethane resin at 100 °C and 100 kPa are normalized to those of PC-PSPs without urethane resin. They are the averages of five measurements taken with each amount of added urethane resin. Figure 18 shows that the luminescence intensity increases with the addition of urethane resin. The normalized intensities are 1.28 and 1.49 at 0.5 g and 1.0 g of urethane resin, respectively. This result also suggests that the urethane resin reduces the rate at which oxygen acts on the dye molecules because the luminescence intensity of PSP is inversely proportional to the oxygen concentration around the dye molecules [1]. 

#### 3.3.5. Frequency Response

**Figure 19:**
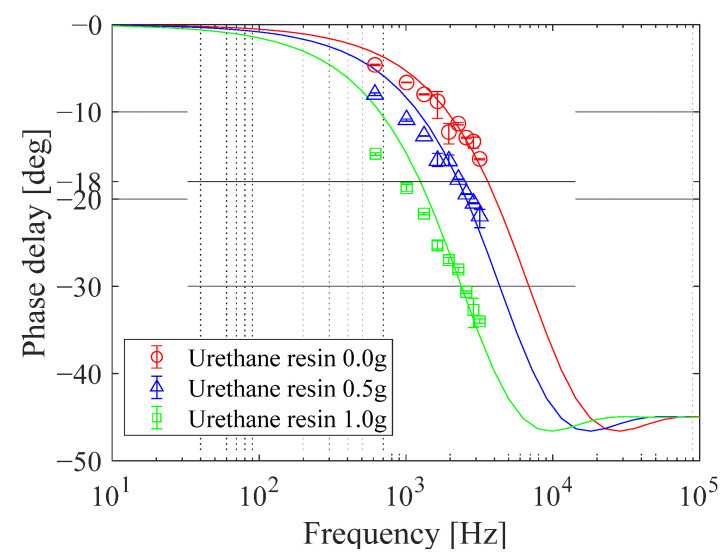
The frequency response diagrams of PC-PSP with different amounts of urethane resin.

The frequency response diagrams of three PC-PSP samples with different amounts of urethane resin are shown in Figure 19. The lines are the approximated PSP frequency response based on the gas diffusion theory [24]. It was found that the more urethane resin is added, the lower the frequency at which the phase difference increases, i.e., the lower the temporal response. Quantitatively, for the urethane resin masses of 0.0 g, 0.5 g, and 1.0 g, the characteristic frequencies at which the phase difference is −18° are 3.6 kHz, 2.4 kHz, and 1.2 kHz, respectively. These results indicate that the addition of urethane resin reduces gas permeability around the dye molecules [1]. The difference between the experimental and theoretical values became larger with the addition of more urethane resin. It suggests that gas permeation into PC-PSP with the addition of urethane resin is more complicated than one-dimensional diffusion in porous media. 

In summary, the addition of urethane resin significantly suppresses the photodegradation of PC-PSPs and increases the luminescence intensity at atmospheric pressure, but slightly reduces pressure sensitivity and time response. These trade-off factors need to be considered when determining the recipe for PC-PSPs to be applied to actual measurements. It should be noted that various polymers can be used as additives for dye solution, as long as they prevent the direct contact of dye with the ambient air. 

## 4. Conclusions

The degradation of polymer-ceramic pressure-sensitive paints (PC-PSP) at high temperatures was characterized to elucidate its mechanism reliably. The PC-PSP consisting of PtTFPP, titania particles and poly IBM were used as a standard sample of PC-PSP. In order to separate the possible degradation factors, we prepared a sample with only the heat treatment and compared its performance with that of the sample without the heat treatment in detail. Characterization was done for luminescence intensity, pressure and temperature sensitivity, luminescence spectra, and frequency response. A two-gate lifetime-based method was employed to evaluate the pressure and temperature sensitivities of the PSP. The glass transition temperatures of the polymers were also investigated through thermal analysis. Then, we tested the addition of a small amount of urethane resin to the thin layer of luminescent dye on the top surface of PC-PSP as a simple additional step to improve the heat resistance of PC-PSP. 

Principal conclusions are summarized as follows:

- The standard PC-PSP has good pressure and temperature sensitivity even at 100 °C. The sensitivity is also almost the same for both.
- While the sensitivity is good, a significant decrease in luminescence intensity occurs at high temperatures. For example, after 1 h of excitation at 100 °C, the luminescence intensity decreases to 0.04. Such a rapid decrease in luminescence intensity at high temperatures makes it difficult to measure the pressure correctly using the intensity-based method of using intensities measured at distant times as reference values. The lifetime-based method should be used if PC-PSP is exposed to high temperatures.
- Subsequent measurements on thermal and photostability as well as luminescence spectra reveal that the main cause of the degradation at high temperature is the photodegradation of PtTFPP due to direct exposure of the dye molecules to atmosphere.
- The effect of glass transition of the polymer does not play a significant role in the lifetime-based PSP measurements, but a very small jump in pressure sensitivity is measured near the glass transition temperature.
- The addition of urethane resin significantly suppresses the photodegradation of PC-PSPs and increases the luminescence intensity at atmospheric pressure, but slightly reduces the pressure sensitivity and time response. These trade-off factors need to be considered when determining the recipe for PC-PSPs to be applied to actual measurements.

## Figures and Tables

**Table 1:** Normalized luminescence intensity of PC-PSP before and after the heat treatment at 20 °C (first and second) and 100 kPa.

	Normalized Intensity [-]	
Heat Treatment	20 °C (First)	20 °C (Second)
Before	1.0	1.0
After	0.43	0.38

**Table 2:** Glass transition temperature of polyIBM and urethane resin.

	Glass Transition Temperature [°C]		
Polymer	Polymer Only	Polymer + PtTFPP	Polymer + PtTFPP (Photodegraded)
PolyIBM	64.7	69.3	69.5
Urethane resin	86.7	94.2	82.7
